# Morphometric variability of neuroimaging features in Children with Agenesis of the Corpus Callosum

**DOI:** 10.1186/s12883-015-0382-5

**Published:** 2015-07-25

**Authors:** Jason Bennett Neal, Christopher G. Filippi, Richard Mayeux

**Affiliations:** Department of Neurology, Columbia University Medical Center, New York Presbyterian Hospital, 710 West 168th St, New York, NY 10032 USA; Department of Neuroradiology, Columbia University Medical Center, New York Presbyterian Hospital, 622 West 168th St, New York, NY USA; Gertrude H. Sergievsky Center, Columbia University, New York, USA

**Keywords:** Agenesis, Dysplasia, Corpus callosum, Brain development, Neuroradiology

## Abstract

**Background:**

Agenesis of the corpus callosum (ACC) is a developmental brain malformation associated with a wide spectrum of structural brain abnormalities and genetic loci. To characterize the diverse callosal morphologies and malformations of brain development associated with ACC, we report on the neuroimaging findings of 201 individuals diagnosed with corpus callosal abnormalities.

**Methods:**

We searched through medical records of individuals seen at New York Presbyterian Hospital between 2002 and 2013 and thought to have ACC. We confirmed 201 individuals meeting criteria and used magnetic resonance imaging to characterize morphological variants of the corpus callosum and associated brain malformations.

**Results:**

The majority of individuals displayed hypoplasia or dysplasia of the corpus callosum (N = 160, 80 %). Forty-one (20 %) displayed complete agenesis of the corpus callosum with other abnormalities, while only 18 (9 %) displayed complete agenesis without associated brain abnormalities. White matter abnormalities were more frequent in hypoplasia or dysplasia group than complete agenesis (28.2 % vs 9.8 %, *p* < 0.05). In contrast, hippocampal abnormalities, colpocephaly, and Probst bundles were significantly more frequent in complete agenesis compared to hypoplasia or dysplasia group.

**Conclusions:**

Collectively, our results underscore the broad diversity of morphological variants of the corpus callosum and associated brain abnormalities in individuals with ACC.

## Background

The corpus callosum (CC) is the primary neuronal fiber tract connecting the two hemispheres of the brain and allows for transfer and integration of sensory, motor, and cognitive information [1]. Anatomically, in a clockwise direction, it is separated into the following four components: rostrum, genu, body, and splenium. Formation of the corpus callosum depends on a series of complex, highly regulated developmental events that begins during gestation and continues until adulthood [2]. The disruption of one or more of these events can result in agenesis of the corpus callosum (ACC), a disorder characterized by the complete or partial loss of one or more components of the corpus callosum [3]. ACC is the most common developmental brain malformation occurring in 1.8 per 10,000 live births and in up to 50 % of individuals who are born with other brain malformations [4].

An estimated 20 % of all cases of ACC are due to genetic causes attributable to single or multiple gene mutations or chromosomal copy number variations [5]. The use of chromosomal microarray technology has recently improved the ability to refine the extent of genomic loci attributable to ACC. Currently, over 30 loci throughout the human genome contain heterozygous loss or gain of function gene products predicted to contain ACC-causative genes [6]. Despite our improved resolution of these loci, the identification of ACC-causative genes has been problematic due, in part, to the broad phenotypic variability of the disorder.

Individuals with ACC display a wide range of morphological architecture of the corpus callosum, which have been described in magnetic resonance imaging studies [7]. The morphological variants of the corpus callosum are divided into three classes based on their appearance on midsagittal MR imaging. Complete agenesis (CAG) is a callosal variant lacking all components of the corpus callosum. Partial agenesis is the absence of some but not all components of the corpus callosum. Hypoplastic corpus callosum is a thin but structurally intact corpus callosum. This three-tier classification system fails to capture the wide range of morphological variability of corpus callosal morphologies. In addition, there is no etiologic basis for this classification system as an equal prevalence of associated brain abnormalities can be seen in each morphological class of abnormal corpus callosum [8]. Recently, a refined classification system of CCAs has been published to account for the deficiencies in the older classification system [9]. The new system segregates callosal variants into classes based on the morphological features that commonly present in consanguineous multiplex families. As probands within the same familial lineage always displayed the same class and often class variant a common genetic etiology might account for similar corpus callosal morphologies [9]. This classification system therefore improves on the previous systems by implicating an etiologic basis for each class of CCA. To date, no studies have implemented this new classification system in the characterization of neuroimaging findings in a cohort of individuals with ACC.

The classification of ACC into specific subtypes defined by imaging would advance future studies aiming to identify the genetic mechanisms giving rise to this disorder. To better characterize the spectrum of callosal variants and associated brain abnormalities in ACC we report a neuroimaging series of 201 individuals with corpus callosal abnormalities.

## Methods

### Participants

Only individuals with MRI imaging were included into this study. One individual had been documented in a prior publication [10]. MRI images were reviewed and reinterpreted by a board-certified neuroradiologist (C.G.F.). Ages corresponded to the age at the initial MRI scan and ranged from 0 to 78 years old. Individuals with medical records that included a diagnosis of ACC at any point in their hospital stay were included in this study. Individuals without MR imaging or with suboptimal image quality, with normal corpus callosum on MRI scans, and secondary causes of callosal anomalies (Chiari malformations, congenital hydrocephalus, hemorrhage, stroke, metabolic disorders, toxin exposure, or infection) were excluded [8].

### Procedures

Approval from the Columbia University Medical Center Institutional Review Board was obtained for searching a database, called “Discovery”, of clinical records of the New York Presbyterian Hospital (NYPH). The database is a clinical research tool developed by the Columbia University Department of Medical Informatics. It compiles clinical information regarding patient demographics, visit history, established diagnoses, procedures performed, imaging studies, and medications stored within the medical records system of every patient admitted at NYPH since 1994. By using this database, researchers have access to vast amounts of data with which to conduct observational clinical research studies. In the current study, the Discovery database was queried for individuals diagnosed with ACC at NYPH between January 2002 and October 2013. Many individuals in this study were initially diagnosed with ACC using low-quality imaging studies such as prenatal screening ultrasound or computed-tomography head scan. Only individuals with 1.5 T strength MR brain imaging and a diagnosis of ACC on a clinical neuroradiology report were included in this study. A separate board-certified pediatric neuroradiologist then reviewed the MR images from all individuals included in this study and designated individuals as having a normal or abnormal corpus callosum.

Individuals with corpus callosal abnormalities were divided into four classes (based on Hanna et al 2011 [9]): hypoplasia, dysplasia, hypoplasia with dysplasia and complete agenesis (CAG). Hypoplasia was sub-divided into 4 subclasses: hypoplasia without dysplasia, apple core, anterior remnant, rudimentary body. Hypoplasia with dysplasia was divided into 2 subclasses: striped and kinked. For statistical analyses corpus callosum abnormalities were divided into two groups, CAG and all other subclasses of agenesis termed “hypoplasia or dysplasia of the corpus callosum”.

MRI images were reviewed for the following abnormalities: (a) hypoplasia or dysplasia of the brainstem, (b) cerebellar anomalies (c) colpocephaly, (d) cysts (interhemispheric and lateral ventricle cysts, subarachnoid cysts, lipomas), (e) cerebral cortical dysplasias (f) Dandy-Walker complex (as previously defined [11]), (g) hippocampal anomalies, (h) neuronal migration anomalies, (i) optic nerve anomalies, (j) Probst bundles, (k) septal anomalies, and (l) white matter anomalies.

### Statistical analysis

Due to small sample sizes, all statistical testing was performed using bi-variate analysis. Statistical comparisons were made between two cohorts: individuals with CAG versus individuals with hypoplasia or dysplasia of the corpus callosum. Student’s *t*-test (two-tailed, unequal variance) was performed to identify significant differences in associated brain malformation, age, or gender. Data are displayed as total number (N), percentages (%), or mean. Data analysis was performed using the Microsoft Excel software platform.

### Ethical considerations

This was a retrospective chart review using information obtained from individual medical health records. Explicit consent for participation in this study was not pursued. However, all protected health information was kept confidential and all authors were blinded to any identifiable information as part of this study.

## Results

**Fig. 1:**
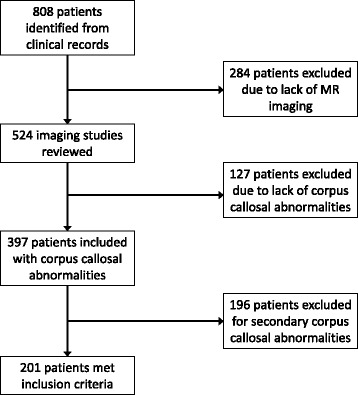
Flow chart of how records of 808 individuals were reviewed from the NYPH medical record system with a diagnosis of agenesis of the corpus callosum between 2003 and 2013. 284 (35 %) patients were excluded due to the lack of MRI imaging, 127 (16 %) because there was no evidence of a corpus callosum abnormality, 196 (24 %) because the abnormality was considered secondary to other causes leaving 201 individuals for detailed review of the characteristics of agenesis of the corpus callosum

Records of 808 individuals from the NYPH medical record system were obtained for individuals diagnosed with ACC between 2003 and 2013. Among these, we excluded 284 (35 %) due to the lack of MRI imaging, 127 (16 %) because there was no evidence of corpus callosum abnormality (due to discrepancy between clinical report and the neuroradiologist’s read), 196 (24 %) because the abnormality was deemed to be secondary to other causes leaving 201 individuals for detailed review of ACC characteristics (Fig. 1). Of those individuals excluded for secondary causes, the most common reasons for exclusion were ischemic injuries to the corpus callosum (N = 61, 31 %), mass lesions or ventriculomegaly leading secondarily to compression of the corpus callosum (N = 57, 29 %), Chiari malformations (N = 10, 5 %), and holoprosencephaly (n = 2, 1 %).

**Fig. 2:**
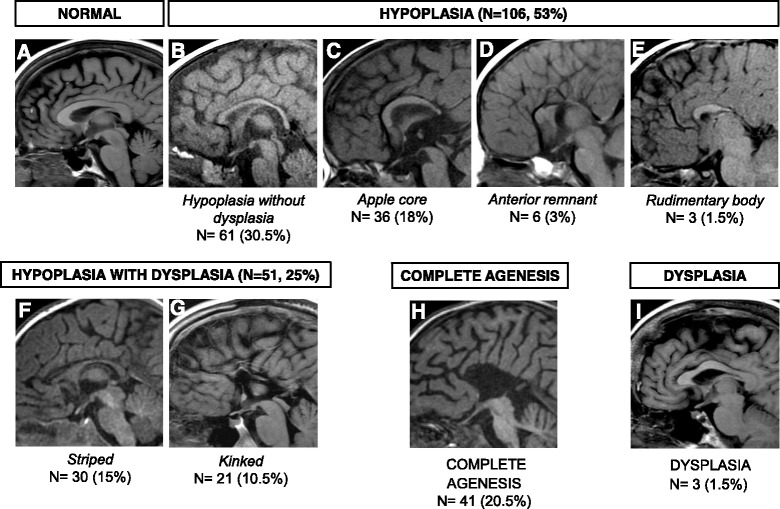
Sagittal MRI images displaying classes and subclasses of corpus callosum abnormalities. Classes of corpus callosum abnormalities denoted in capital letters, subclasses in italics. Number and percentage of patients are displayed for each subclass. **a** Normal brain (**b**) hypoplasia without dysplasia (**c**) apple core (**d**) anterior remnant (**e**) rudimentary body (**f**) striped (**g**) kinked (**h**) complete agenesis (**i**) dysplasia

All four classes of corpus callosum abnormalities were identified with hypoplasia being the most frequent (N = 106, 53 %) (Fig. 2). The most frequent subclass of hypoplasia was without dysplasia (N = 61, 30.5 %, Fig. 2b), followed by apple core (N = 36, 18 %, Fig. 2c), and anterior remnant (N = 6, 3 %, Fig. 2d). Three individuals (1.5 %) displayed a hypoplastic corpus callosum with rudimentary body but absence of a genu, rostrum, and splenium (Fig. 2e). This variant is not well characterized by existing subclasses and therefore represents a new subclass of hypoplasia that we named “rudimentary body abnormality”. Hypoplasia with dysplasia was the second most frequent class of corpus callosum abnormality (N = 51, 25 %). Striped abnormality was more common (N = 30, 15 %, Fig. 2f) than kinked (N = 21, 10.5 %, Fig. 2g). CAG was the third most frequent class of corpus callosum abnormality (N = 41, 20.5 %, Fig. 2h) and dysplasia was the least frequent class (N = 3, 1.5 %, Fig. 2i).

Collectively, the average age of the study cohort was 45 months (3.75 years) old with a median age of 6 months and mode of 0 months. The ages of individuals with hypoplasia or dysplasia were significantly greater (4.31 ± 10.3 years) than individuals with CAG (1.43 ± 3.09 years) (*p* < 0.01) (Table 1). However, median ages for both CAG (0.04 years) and the hypoplasia or dysplasia group (0.5 years) were less than one year. 39.6 % of the entire study sample was female. There was no significant difference in sex between CAG (41.5 % female) and hypoplasia or dysplasia (38.8 % female) (*p* = 0.751) (Table 2).

**Table 1:** Ages of individuals with complete agenesis and hypoplasia or dysplasia

	Complete agenesis	Hypoplasia or dysplasia
Average age in months (years)^a^	17.2 (1.43)	51.7 (4.31)
Median age in months (years)	0.5 (0.04)	6 (0.5)
Standard deviation of age in months (years)	37.1 (3.09)	124 (10.3)

^a^ *p* < 0.01

**Table 2:** Sex of individuals with Complete agenesis and Hypoplasia or dysplasia

	Complete agenesis	Hypoplasia or dysplasia
Percentage female	41.5	38.8

*p* = 0.751

Individuals within each class and subclass of corpus callosum abnormality displayed a wide range of associated central nervous system defects (Tables 3 and 4). Compared to individuals with hypoplasia or dysplasia of the corpus callosum, individuals with CAG showed a significantly greater frequency of isolated corpus callosum abnormalities (without associated central nervous system defects), hippocampal anomalies, Probst bundles, and colpocephaly. In contrast, the hypoplasia or dysplasia group showed a significantly greater frequency of white matter anomalies compared to CAG. Individuals with hypoplasia or dysplasia showed no significant differences in the frequency of brainstem anomalies, neuronal migration anomalies, dysplasias of the cerebral cortex, cerebellar anomalies, cysts, Dandy-Walker complex, optic nerve anomalies, or septal anomalies compared to individuals with CAG (Table 3).

**Table 3:** Comparison of associated brain malformations among individuals with hypoplasia or dysplasia and complete agenesis of the corpus callosum

Associated brain malformation	Complete agenesis	Hypoplasia or Dysplasia
Brainstem anomalies	4(9.76 %)	7(4.38 %)
Cerebellar anomalies	5(12.2 %)	8(5.00 %)
Colpocephaly^a^	13(31.7 %)	5(3.13 %)
Cysts	6(14.6 %)	17(10.6 %)
Dysplasia of the cerebral cortex	2(4.88 %)	8(5.00 %)
Dandy-Walker complex	5(12.2 %)	26(16.3 %)
Hippocampal anomalies^b^	11(26.8 %)	15(9.38 %)
Isolated callosal anomaly^c^	18(43.9 %)	44(27.8 %)
Neuronal migration anomalies	14(34.15 %)	35(21.9 %)
Optic nerve anomalies	1(2.44 %)	5(3.13 %)
Probst bundles^a^	18(43.9 %)	1(0.63 %)
Septal anomalies	0(0 %)	16(10.0 %)
White matter anomalies^c^	4(9.76 %)	46(28.8 %)

^a^ *p* < 0.001, ^b^ *p* < 0.01, ^c^ *p* < 0.05

**Table 4:** Associated brain malformations identified on MRI imaging

	Hypoplasia without dysplasia	Apple core	Anterior remnant	Rudimentary Body	Striped	Kinked	Complete agenesis	Dysplasia
Cysts	7 (39.4 %)	2 (8.70 %)	3 (13.0 %)	0 (0 %)	5 (21.7 %)	0 (0 %)	6 (26.1 %)	0 (0 %)
Brainstem anomalies	1 (9.09 %)	1 (9.09 %)	1 (9.09 %)	0 (0 %)	4 (36.4 %)	0 (0 %)	4 (36.36 %)	0 (0 %)
Cerebellar anomalies	2 (15.6 %)	0 (0 %)	0 (0 %)	0 (0 %)	4 (30.8 %)	2 (15.6 %)	5 (38.5 %)	0 (0 %)
Colpocephaly	1 (5.56 %)	0 (0 %)	2 (11.1 %)	1 (5.56 %)	0 (0 %)	1 (5.56 %)	13 (72.2 %)	0 (0 %)
Cortical dysplasia	1 (10 %)	6 (60 %)	0 (0 %)	0 (0 %)	1 (10 %)	0 (0 %)	2 (20 %)	0 (0 %)
Dandy-Walker complex	8 (25.8 %)	6 (19.4 %)	1 (3.23 %)	0 (0 %)	9 (29.0 %)	2 (6.45 %)	5 (16.1 %)	0 (0 %)
Hippocampal anomalies	2 (7.69 %)	3 (11.5 %)	4 (15.4 %)	1 (3.85 %)	3 (11.5 %)	2 (7.69 %)	11 (42.3 %)	0 (0 %)
Neuronal migration anomalies	12 (24.5 %)	9 (18.4 %)	2 (4.08 %)	2 (4.08 %)	5 (10.2 %)	5 (10.2 %)	14 (28.6 %)	0 (0 %)
Optic nerve anomalies	0 (0 %)	0 (0 %)	0 (0 %)	0 (0 %)	4 (66.7 %)	1 (16.7 %)	1 (16.7 %)	0 (0 %)
Probst bundles	0 (0 %)	0 (0 %)	1 (5.26 %)	0 (0 %)	0 (0 %)	0 (0 %)	18 (94.7 %)	0 (0 %)
Septal anomalies	7 (43.8 %)	3 (18.8 %)	0 (0 %)	0 (0 %)	3 (18.8 %)	2 (12.5 %)	0 (0 %)	1 (6.25 %)
White matter anomalies	14 (28.0 %)	8 (18.0 %)	1 (2.00 %)	2 (4.00 %)	14 (28.0 %)	5 (10.0 %)	4 (8.00 %)	1 (2.00 %)

## Discussion

To address the variability of corpus callosum abnormalities we present a neuroimaging series of 201 individuals with ACC. Our data mirrors previously published data indicating that nearly one-in-five individuals diagnosed with ACC at a tertiary care hospital have complete agenesis or CAG [3]. These individuals were younger than those with hypoplasia or dysplasia possibly because CAG is more easily identified on prenatal ultrasound screening than in the hypoplasia or dysplasia group [12, 13]. However, there is selection bias inherent because our study did not include prenatal cases of ACC, of which up to 42.4 % are terminated prior to birth [14].

Colpocephaly was more frequent in CAG than in the hypoplasia or dysplasia group. It is caused by decreased white matter in the occipital cortex leading to secondary expansion of the posterior horns of the lateral ventricles [8, 15]. The preservation of myelinated callosal tracts in the hypoplasia or dysplasia group likely provides structural integrity preventing posterior expansion of the lateral ventricles. Probst bundles were more frequent in CAG than in the hypoplasia or dysplasia group. Probst bundles are longitudinal axonal fiber tracts of the corpus callosum that have failed to cross the midline into the contralateral hemisphere and form ectopic fiber bundles along the dorsomedial lateral ventricular surface [15, 16]. Increased thickness of Probst bundles, given the absence of crossing fibers in CAG, is likely to account for its visualization on MRI. Hippocampal anomalies were more frequent in CAG versus in the hypoplasia or dysplasia group. Developmental studies in mice demonstrate that incipient axonal collaterals from the hippocampal primordium serve as “guideposts” for subsequent callosal axons to cross into the contralateral hemisphere [16, 17]. Therefore, disruptions in hippocampal development may indirectly cause early and severe disruptions in callosal development leading to CAG. White matter anomalies were also more frequent in the hypoplasia or dysplasia group versus CAG. Initial callosal tracts are established prior to the beginning of myelination thereby mechanisms guiding myelination would not be expected to contribute to widespread corpus callosum abnormalities [15]. However, CAG has been reported with cholesterol biosynthesis aberrations suggesting that white matter abnormalities can lead to CAG as well [18].

The use of the refined classification system by Hanna et al [9] provides many advances in the investigation of the causes of ACC. The heterogeneity of callosal morphologies is more accurately described by the inclusion of subclasses of corpus callosum abnormalities and almost sufficient to characterize our entire cohort. One exception was the *rudimentary body abnormality*, which lacks rostral callosal components. As rostral and caudal regions of the corpus callosum are thought to be regulated by separate developmental mechanisms, this subclass likely has a unique genetic mechanism [16].

## Conclusion

These results further support a significant heterogeneity in the spectrum of corpus callosum morphologies and associated brain malformations in individuals with ACC. The improved accuracy with which to classify the diverse morphologies of corpus callosal abnormalities might enhance the robustness of genetic studies by allowing similar phenotypes to be grouped together. The combination of this morphology based classification system with a separate classification system based on genetic variants should allow for further elucidation of the causes of ACC.
